# Relationship between COVID-19 Pandemic-Related Life Behavior, Dispositional Mindfulness, and Psychological Health: Evidence from a Sample of Japanese Working Adults

**DOI:** 10.3390/ijerph20105873

**Published:** 2023-05-19

**Authors:** Hiromitsu Miyata, Kaho Yamasaki, Noh ChaeEun, Haruyuki Ishikawa

**Affiliations:** Faculty of Letters, Arts and Sciences, Waseda University, 1-24-1 Toyama, Shinjuku-ku, Tokyo 162-8644, Japan

**Keywords:** COVID-19 pandemic, altered lifestyle, dispositional mindfulness, psychological health, moderation, Japanese workers

## Abstract

The present study investigated how altered daily life behavior and its self-evaluation associated with the coronavirus disease 2019 (COVID-19) pandemic relate to psychological health in Japanese working adults, and how such relationships may be moderated by dispositional mindfulness. A total of 1000 participants completed an online survey comprising questions on how they used time and self-evaluated life behavior before and during the pandemic, as well as scales on mindfulness and psychological health. The results revealed that after the pandemic, participants spent significantly more time at home and using a PC/smartphone. They were also more likely to perceive frequent exposure to COVID-19-related media reports and less likely to find their work going well. Many of these variables were significantly correlated with lower psychological health. Moreover, hierarchical multiple regression analyses revealed the moderating effects of mindfulness, such that the perceived frequency of exposure to pandemic-related media reports and poorer views that work was going well were less likely to predict lower psychological health when mindfulness was high. These findings suggest that altered daily life behavior and its self-evaluation after the pandemic are associated with deteriorated psychological health, but that mindfulness can serve as a protective factor against psychological distress among Japanese workers.

## 1. Introduction

The concept of mindfulness is typically defined as a mental attitude in which one is paying attention to the present moment while perceiving reality as it is without evaluation or judgment [1]. The idea of mindfulness is deemed be originated from the Eastern tradition of contemplative and/or mind–body practices, which involve different meditations such as focused-attention and open-monitoring meditations [2,3]. Mindfulness has been investigated as both a state (i.e., temporary condition) and a trait (i.e., stable personality or characteristic). Regarding state mindfulness, empirical studies on mindfulness-based interventions have demonstrated that such interventions can enhance state mindfulness to result in desirable psychological health outcomes in patients with chronic pain [1,4], in individuals with a risk of depression relapse [5], and in individuals with a wide variety of psychosomatic symptoms or disorders (for an overview, see [6]). In a nonclinical and/or noninterventional context, mindfulness is also regarded as a psychological trait that is significantly related to desirable psychological health outcomes, regardless of whether an individual engages in the habitual practice of mindfulness-based interventions [7,8]. Such mindfulness as an inherent trait of humans is referred to as dispositional mindfulness [8,9]. Dispositional mindfulness is deemed as a psychological construct having multiple facets, including observing the internal and external world, verbally describing one’s experience, acting with awareness of the present moment, nonjudgmental attitude toward one’s inner experience, and nonreactivity to one’s inner experience [10]. In a Japanese population of nonpractitioners of mindfulness, Sugiura and Sugiura [11] reported that multiple dimensions of dispositional mindfulness, i.e., the description and nonjudgment of inner experience, significantly moderated the relationship between income levels and psychological well-being.

The present study focuses on whether and how dispositional mindfulness may serve as an effective protective factor during the coronavirus disease 2019 (COVID-19) pandemic. In 2020, the emergence of the COVID-19 pandemic rapidly and drastically changed physical/social environments and life behavior around the world (for an overview, see [12,13,14]). Before effective vaccinations against COVID-19 were developed, staying at home was regarded as the optimal strategy to prevent the spread of infection. Governments, therefore, stated that people should refrain from going out as much as possible. This situation led to significant changes in how people spent their time and behaved in daily life, including reductions in physical activity and increases in sedentary behaviors such as sitting and/or screen times [14]. The pandemic also evoked changes in workstyles, such as increased remote work or teleworking, i.e., working at home instead of at an office, which significantly reduced communication with colleagues [15]. In addition, the massive levels of unemployment caused by employers’ financial difficulties affected the lives of millions of individuals and families during the pandemic period [16]. In Japan, the Ministry of Land, Infrastructure, and Transport conducted a questionnaire survey on the daily life behavior of 12,872 Japanese workers before and during the COVID-19 pandemic [17,18]. The survey was conducted in August 2020 by developing original question items and revealed changes in both time use (e.g., time spent at home, sleeping time, etc.) and how individuals evaluated daily life behavior (e.g., communication with colleagues or family) after the pandemic.

These altered environments and daily life behavior have been suggested to have a significant impact on psychological health outcomes across the world [19,20,21,22]. For example, Alzueta et al. [23] included a sample of 59 countries from 5 continents and reported moderate to severe depression and anxiety symptoms after the COVID-19 outbreak. Meyer et al. [14] reported that decreased physical activities and increased screen time were significantly associated with higher perceived stress and depression and greater loneliness in a US sample. Targa et al. [24] reported that sleep quality in a Spanish sample worsened during the initial months of the pandemic in 2020 and that this change was significantly associated with an increase in negative mood. Exposure to a vast amount of COVID-19-related information from mass media, news sites, and/or social media, as termed an “infodemic”, can also impact psychological health [25,26]. Yomoda [27] analyzed Japanese texts posted on Twitter from January to March 2020 and reported that not only fear of being infected with the coronavirus and restrictions in daily activities but also the anxiety caused by the news media, such as TV programs, increased considerably during the initial weeks of the COVID-19 outbreak. In addition, the increased prevalence of remote work was reported to lead to increased psychological distress, isolation, and dissatisfaction with work among workers [15,28]. Moreover, the loss of work after the pandemic is suggested to have led to feelings of existential loss, anxiety, and fear [16].

Given these changes in daily life behavior and deteriorated psychological outcomes related to the COVID-19 pandemic, it is important to examine potential protective factors such as mindfulness for psychological health during the pandemic period [29,30]. Because mindfulness entails observing the moment-to-moment experience with acceptance [1,10], such mental attitudes should help refrain from overreacting to altered and/or uncertain daily life situations such as time use and the self-evaluation of daily life behavior during emergencies such as the COVID-19 outbreak. Therefore, mindfulness can protect individuals against deteriorated psychological health during the pandemic, regardless of whether an individual engages in the habitual practice of mindfulness or meditation. Empirical data obtained during the COVID-19 pandemic overall concur in finding the protective roles of mindfulness-based interventions for psychological health [31]. Al-Refae, Al-Refae, Munroe, Sardella, and Ferrari [32] showed that a 4-week mindfulness-based intervention using a smartphone improved self-compassion and emotion regulation during the pandemic period. Several empirical studies from nonclinical contexts also suggested associations between dispositional mindfulness and desirable psychological outcomes during the pandemic [33,34]. For example, Sbrilli, Haigler, and Laurent [34] reported that a reduction in dispositional mindfulness mediated the relation between intolerance to uncertain experiences during the pandemic and psychological symptoms in pregnant women and new mothers in the US.

In these research contexts, the present study focused on a Japanese population of working adults. Investigating this relatively understudied population should make significant sense because the cultural background of Japan, which largely originated from Buddhism and/or Zen, is suggested to involve rich psychological elements of mindfulness, such as cultivating the awareness of experiences in the present moment and communing with nature with awe and gratitude [35,36]. In addition, the nature of mind–body practices such as martial arts originated in Japan are suggested to converge with those of Zen as well as mindfulness [37]. Miyata et al. [37] reported that the period and frequency of martial arts practice based on Japanese traditions were associated with higher dispositional mindfulness and more desirable psychological health outcomes. In addition, in a Japanese population with no daily practice of martial arts or mindfulness, higher dispositional mindfulness was found to be associated with better psychological health [7]. These perspectives and the empirical data lead to an assumption that studying this population could uncover how the psychological dispositions that have underlain Japanese people, even those with no explicit reference to mindfulness, could help protect psychological health during emergencies.

Accordingly, the present study aimed to clarify post-pandemic changes in daily life behavior including time use and the self-evaluation of life behavior, and how such changes were associated with psychological health among Japanese working adults during the relatively early stages of the COVID-19 outbreak. Based on these analyses, we further aimed to uncover the potential role of dispositional mindfulness in moderating these associations. The results of the survey on daily life behavior conducted by Japan’s government [17] or Japanese versions of psychological scales on fear of COVID-19 [38] were not published at the time of the present study. We, therefore, developed original question items on COVID-19-related daily life behavior, assuming that both time use and the self-evaluation of daily life behavior should have significantly changed after the pandemic. Based on the suggested impacts of the COVID-19 pandemic on mental health, we hypothesized that both these changes would have detrimental effects on the psychological health of this population. Furthermore, given the preceding findings suggesting the protective roles of mindfulness for psychological health during the pandemic, we also hypothesized that dispositional mindfulness would moderate the relationships between both time use and the self-evaluation of daily life behavior and psychological health outcomes.

## 2. Methods

### 2.1. Participants and Procedures

A total of 1000 Japanese working adults (309 females and 691 males; age range 20–59 years; mean age 46.8 years, standard deviation (SD) 9.0) were included in the study. After log transformation (base e) of the time to complete the survey (mean 28.2 min, SD 62.3), outliers were removed at two SD*s* above and below the mean; that is, either incorrect or unusual response patterns were suspected for those participants whose answer times were extremely short or long [39,40]. After excluding 56 participants whose time to complete the survey exceeded two SDs above the mean, data for 944 participants (293 females and 651 males; age range 20–59 years; mean age 46.8 years, SD 9.1) were subjected to analysis. These participants’ demographic and other basic characteristics are summarized in Table 1. We included participants across a wide range of age groups: 5.3% in their twenties, 15.1% in their thirties, 32.7% in their forties, and 46.8% in their fifties. No students were included in the survey. Additionally, no participants were unemployed at the time of the survey. Regarding the household income level, JPA 2,000,000, 5,000,000, and 9,000,000 corresponded to USD 18,957, 47,393, and 85,308, respectively (as of 28–29 September 2020, on which USD 1 corresponded to JPA 105.5). In 2020, the mean household income in Japan was JPA 5,643,000 for all households, JPA 3,329,000 for households with member(s) 65 years of age or above, JPA 8,135,000 for households with children, and JPA 6,859,000 for the other households [41]. Besides the demographic variables, 8.5% of the participants reported practicing mindfulness and/or meditation on a daily basis. All the participants were monitors of an academic research system run by NEO Marketing Inc., Tokyo, Japan. NEO Marketing runs multiple marketing support businesses including marketing research, digital marketing, branding services, etc. Among these businesses is the academic research system, which runs various academic questionnaire surveys upon request from universities, research institutions, etc. This system had more than 19 million Japanese registered monitors as of 2020, who were not employees of the company and had provided their demographic information including sex, age, and employment status, and agreed to cooperate with various online questionnaire surveys. The participants for the present survey were selected by the company from the pool of these registered individuals, according to the criteria that they were 20–59 years old and not unemployed at the time of the survey. Within the system, registered monitors accumulated points as they completed different online surveys, after which they were able to exchange the points for monetary rewards.

All data were collected on the original online platform of the research system. To ensure that there were no missing data, the survey was not considered complete until the participants answered all question items and scales. Before determining the sample of 1000 participants, an additional 2.0% of individuals were excluded, all of whom had agreed to cooperate and completed the survey. For question items regarding time use, individuals were excluded if they gave an answer of “more than 24 h per day” or gave identical answers to all the items. For the questions regarding the self-evaluation of daily life behavior, individuals were excluded if they checked identical options to all the items. Regarding the psychological scales, individuals were excluded if they checked identical options to all the items for the scale on dispositional mindfulness, or checked the identical options throughout the other three scales. Data collection was conducted on 28–29 September 2020. Except for a restriction to complete the survey within these dates, there were no limitations regarding when or where the participants should complete the survey.

### 2.2. Measures

#### 2.2.1. COVID-19-Related Daily Life Behavior

The survey included original question items on the daily life behavior associated with the pandemic, which aimed to examine the latest changes after the pandemic. These items included both time use and the self-evaluation of daily life behavior, with the latter involving both measures directly related to COVID-19 and other daily life routines. For each question item, the participants were required to report both a normal value from April 2020 to the date of the survey (during the COVID-19 pandemic), and a normal value from October 2019 to March 2020 (before the COVID-19 pandemic began to influence their daily life), as far as they could recall at the time of the survey.

In order to create these question items, we conducted a preliminary survey in August 2020, in which we asked seven adults to freely describe any specific post-pandemic lifestyle changes and the impacts of such changes on psychological health that they either recognized or heard from workers around them. These individuals were undergraduate students belonging to a university located in Tokyo (six females and one male; age range 20–22 years), who were neither participants of the main study nor employees of NEO Marketing Inc. Given that the sample of the main study included Japanese workers who were not students, these individuals were required to do their best to describe the general status of Japanese workers at the time of the survey. The questions for the main survey were developed with reference to the descriptions provided in this preliminary survey, all of which were regarded to fall into either of the abovementioned two categories.

Regarding time use, the participants were asked to answer the following 10 items in hours per day up to one decimal place (phrases in italics are those directly translated into English from the original Japanese phrases): (1) *time spent at home (except for sleeping time)*; (2) *time spent indoors other than at home (such as an office, except for sleeping time)*; (3) *time spent outdoors (except for sleeping time)*; (4) *sleeping time*; (5) *time spent using a PC (e.g., desktop PC, laptop, tablet)*; (6) *time spent using a smartphone*; (7) *time spent engaging with social media (e.g., Facebook, Twitter, Instagram)*; (8) *time spent engaging in work at home (remote work)*; (9) *time spent engaging in work at places other than at home (e.g., at an office)*; and (10) *time spent engaging in activities other than work (e.g., sports, art, hobbies, leisure activities)*.

Regarding the self-evaluation of daily life behavior, participants were presented with eight sentences and asked to evaluate the extent to which each sentence was true for them. These question items used a 10-point Likert scale from “1 (not true at all)” to “10 (extremely true)”, and the score for each item was analyzed as a single-item measure. The sentences for these question items were as follows (sentences in italics are those directly translated into English from the original Japanese sentences): (1) *I feel that I may be infected with COVID-19* (hereinafter referred to as “anticipation of infection”); (2) *I feel that I am frequently exposed to COVID-19-related media reports* (“media exposure”); (3) *I feel that I am living a well-regulated life (My lifestyle is not disturbed)* (“well-regulated life”); (4) *I feel that I am getting enough sleep* (“enough sleep”); (5) *I feel that my work is going well* (“smoothness of work”); (6) *I feel that my activities other than work (e.g., sports, art, hobbies, leisure activities) are going well* (“smoothness of nonwork activities”); (7) *I feel that I am maintaining sufficient communication at work* (“communication at work”); and (8) *I feel that I am maintaining sufficient communication with my family* (“communication with family”).

#### 2.2.2. Dispositional Mindfulness

To examine dispositional mindfulness and psychological health, we used the established Japanese versions of psychological scales. First, we used the Five Facet Mindfulness Questionnaire (FFMQ; [10]), which is one of the most frequently used measures of dispositional mindfulness. The FFMQ has 39 items, each rated on a 5-point scale ranging from “1 (never or very rarely true)” to “5 (very often or always true)”. The items fall into one of the following five facets (subscales): *observing* (8 items); *describing* (8 items); *acting with awareness* (8 items); *nonjudging of inner experience* (8 items); and *nonreactivity to inner experience* (7 items). The score for each facet is calculated by summing these items, and a total score is also calculated. Cronbach’s α for the present sample was 0.79 for the total FFMQ score and 0.78–0.86 for the subscale scores. The present study used the Japanese version of the FFMQ developed by Sugiura, Sato, Ito, and Murakami [42]. The participants were instructed to look back on the post-pandemic period from April 2020 to the time of the survey and to select the option that best described their normal status.

#### 2.2.3. Perceived Stress

Next, we used the Perceived Stress Scale (PSS; [43]), a standard measure that assesses the degrees of perceived stress. Sumi [44] developed the Japanese version of the PSS, which was used in the present survey. The PSS is composed of 14 sentences as items, each pertaining to a situation in one’s life that can be appraised as stressful. Each item is rated on a 5-point scale, from “0 (never)” to “4 (very often)”. Scores from all items are summed to yield a total score. Consistent with the FFMQ, the participants were instructed to report their normal feelings and thoughts during the period from April 2020 to the time of the survey. Cronbach’s α for the present sample was 0.82.

#### 2.2.4. Anxiety

The State–Trait Anxiety Inventory (STAI; [45]) is a commonly used psychological scale that separately measures state anxiety (STAI-S) and trait anxiety (STAI-T). Shimizu and Imae [46] developed the Japanese version of the STAI. We used the STAI-T to assess trait anxiety, which consists of 20 items rated on a 4-point scale from “1 (almost never)” to “4 (almost always)”. The total score is calculated by summing these items. We instructed the participants to report their normal psychological status during the period from April 2020 to the time of the survey, consistent with the scales above. Cronbach’s α for the present sample was 0.91.

#### 2.2.5. Depression

The Center for Epidemiologic Studies Depression Scale (CES-D; [47]) is a widely used scale that measures self-reported levels of depressive symptoms. We used the Japanese version of the CES-D developed by Shima, Shikano, Kitamura, and Asai [48]. The CES-D is composed of 20 self-evaluative sentences as items. For each item, participants endorse one of four alternatives based on the status of their own body and mind within the latest week, i.e., A: rarely or none of the time (less than 1 day); B: some or a little of the time (1–2 days); C: occasionally or a moderate amount of time (3–4 days); and D: most or all of the time (5–7 days). Each response is scored from 0 to 3, and a total score is calculated. Cronbach’s α for the present sample was 0.91.

### 2.3. Statistical Analyses

Statistical analyses were conducted using HAD version 17 [49]. HAD is a download-free software that runs on the Microsoft Excel VBA. Besides the fact that it is available for free (https://osf.io/32cyp/ (accessed on 1 April 2023)), HAD covers major statistical analyses including descriptive statistics, correlational analysis, analysis of variance, and moderated multiple regression analysis and visualization of the interactions. Regarding the question items regarding COVID-19-related daily life behavior, the means and *SD*s for the pre- and post-pandemic periods were calculated, respectively, after which, comparisons between these periods were made by using paired *t*-tests. For the psychological scales, after calculating the total and subscale scores, zero-order correlations (Pearson’s *r*) between the total/subscale scores were analyzed. We also examined the correlations between post-pandemic daily life behavior and characteristics of participants including age, household income level, and number of people per household, which were either interval or ordinal scales. Because household income level and the number of people per household were ordinal scales, Spearman’s rank correlation coefficients (*r_s_*) were used for these measures. Next, we examined the correlation coefficients (*r*) between the outcomes from the questions regarding COVID-19-related daily life behavior and the total scores from each psychological scale. In addition to including daily life behavior during the post-pandemic period, differences in these variables between the pre- and post-pandemic periods were used for the analyses to assess whether and how changes in daily life behavior were associated with psychological status.

Furthermore, hierarchical multiple regression analyses were conducted to examine the potential moderating effects of dispositional mindfulness on the relations between COVID-19-related daily life behavior and psychological health outcomes. Regarding the variables on daily life behavior, the regression models included answers based on the post-pandemic period because these answers yielded more apparent correlations with psychological status than the pre–post differences. In fact, answers based on the pre-pandemic period are likely to be more susceptible to the influence of a recall bias than answers based on the latest period [50]. Models with the total scores from the PSS, STAI-T, and CES-D as dependent variables were examined separately. A proposed moderator for all these models was the total scores from the FFMQ. Because we examined the interactions in these models, all independent variables were centered for these analyses.

In Step 1, we included demographic and other control variables, involving sex, age, marital status, employment status, household income level, and number of people per household, as well as whether the participant engaged in the daily practice of mindfulness/meditation. Among the employment status categories, self-employed/family worker was excluded because the variance inflation factor (VIF) values, a measure of multicollinearity, were larger than 10 when the category was included in the models. In Step 2, we included the selected variables on COVID-19-related daily life behavior to examine their main effects. These variables were selected in order to examine the moderating roles of dispositional mindfulness for the dimensions of daily life behavior that had undergone relatively large changes after the pandemic. Regarding time use, changes in the time spent at home and engaging in work at home between the pre- and post-pandemic periods met a standard of at least a small effect size (Cohen’s *d* > 0.20; [51]). Among these measures, the time spent engaging in work at home failed to show significant correlations with the total scores from the psychological scales and thus was not included in the models. Instead, the time spent using a smartphone and engaging with social media were included in the models because these measures showed relatively apparent correlations with mindfulness and/or psychological health outcomes. Regarding the self-evaluation of daily life behavior, changes in the anticipation of infection, media exposure, and smoothness of work met the same standard as above. These three measures showed significant correlations with mindfulness and/or psychological health outcomes and were therefore included in the models. The total FFMQ scores were also included in Step 2. The time spent engaging with social media, the anticipation of infection, media exposure, and smoothness of work, as well as the total FFMQ scores, significantly predicted one or more psychological health outcome(s) in Step 2; therefore, Step 3 involved the interactions between these variables on daily life behavior and FFMQ scores. With regard to the significant interactions in Step 3, post hoc simple slope analyses were further applied to examine the effect of FFMQ scores at 1 *SD* above and below the mean.

## 3. Results

### 3.1. COVID-19-Related Daily Life Behavior and Pre–Post Comparisons

Table 2 shows both time use and the self-evaluation of daily life behavior during the pre- and post-pandemic periods. Statistically significant changes were observed for all these measures, including altered time use, such as an increase in the time spent at home; a decrease in the time spent indoors other than at home (e.g., office work); an increase in the time spent using a smartphone and engaging with social media; and an increase in the time spent working at home (remote work), as well as apparently less desirable self-evaluation of daily life behavior such as an increase in the anticipation of infection, an increase in COVID-19-related media exposure, and a decline in positive views toward the smoothness of work. Some measures on the self-evaluation of life behavior showed more desirable outcomes after the pandemic, including a better-regulated life, more sufficient sleep, and better communication with family. Thus, multiple aspects of the participants’ time use and self-evaluation of daily life behavior changed significantly after the COVID-19 pandemic, even though a limited number of measures met a standard of at least a small effect size (Cohen’s *d* > 0.20; [51]).

### 3.2. Scores from Psychological Scales and Correlations between Scales

The total and all subscale scores from the FFMQ showed statistically significant negative correlations with the total scores from the PSS, STAI-T, and CES-D, except for a nonsignificant correlation between the observing subscale of the FFMQ and the PSS (Table 3). The total scores from the PSS, STAI-T, and CES-D were significantly and positively correlated with each other. Thus, higher dispositional mindfulness was significantly associated with more desirable psychological health outcomes.

### 3.3. Correlations between Characteristics of Participants and Daily Life Behavior

A significant positive correlation was found between household income level and the number of people per household (*r_s_* = 0.369, *p* < 0.001). Age was significantly and positively correlated with household income level (*r_s_* = 0.122, *p* < 0.001) and was significantly negatively correlated with the number of people per household (*r_s_* = −0.079, *p* = 0.016). Table 4 summarizes the correlations between age, household income level, the number of people per household, and post-pandemic daily life behavior. Age showed significant negative correlations with sleeping time, the time spent using a smartphone, the time spent engaging with social media, the smoothness of nonwork activities, etc. Household income level showed significant negative correlations with the time spent at home and sleeping time, and significant positive correlations with the time spent indoors other than home, the time spent using a PC, and the time engaging in work at home. Household income level also showed significant positive correlations with the self-evaluation of life behavior including the smoothness of work, communication at work, communication with family, etc. Similar correlations were found for the number of people per household, although some measures (e.g., time spent using a PC) showed opposite significant correlations. Thus, these characteristics of participants were significantly associated with post-pandemic measures on COVID-19-related daily life behavior.

### 3.4. Correlations between COVID-19-Related Daily Life Behavior and Psychological Scales

Analyses based on post-pandemic life behavior revealed statistically significant correlations between time use and psychological status, including positive correlations between the time spent using a smartphone and engaging with social media and less desirable psychological health outcomes (Table 5). More apparent significant correlations were found between the post-pandemic self-evaluation of life behavior and psychological status, such that greater degrees of anticipation of infection with COVID-19, more frequent exposure to pandemic-related media reports, and poorer views toward the progress of the participants’ work were associated with lower dispositional mindfulness and/or psychological health. Regarding analyses based on pre- and post-pandemic changes, no statistically significant correlations were found between time use and psychological status (Table 5). Some measures regarding changes in the self-evaluation of life behavior, such as the smoothness of work, were significantly correlated with mindfulness and/or psychological health outcomes; however, these correlations were less apparent than when post-pandemic measures were included.

### 3.5. Moderating Effects of Mindfulness

In all three hierarchical multiple regression models, the increase in the coefficient of determination (Δ*R*^2^) from Step 1 to Step 2 was statistically significant (PSS: Δ*R*^2^ = 0.440, *p* < 0.001; STAI-T: Δ*R*^2^ = 0.408, *p* < 0.001; CES-D: Δ*R*^2^ = 0.330, *p* < 0.001). Models involving the PSS (Δ*R*^2^ = 0.012, *p* < 0.001) and CES-D (Δ*R*^2^ = 0.020, *p* < 0.001) also showed a significant increase in *R^2^* values from Step 2 to Step 3 (Table 6). VIF values for the independent variables in Step 3 were between 1.050 and 3.703 in these three models, indicating the absence of serious multicollinearity. In Step 3, an interaction between media exposure and FFMQ scores significantly predicted PSS scores (*β* = −0.097, *p* < 0.001). Interactions between the smoothness of work and FFMQ scores also significantly predicted PSS (*β* = 0.085, *p* < 0.001), STAI-T (*β* = 0.050, *p* = 0.046), and CES-D (*β* = 0.148, *p* < 0.001) scores (Table 6).

**Figure 1:**
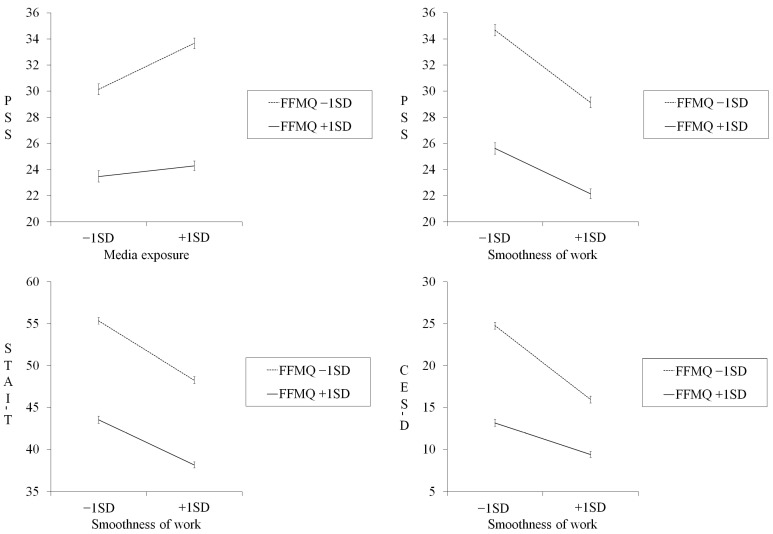
Results of simple slope analyses showing moderating effects of dispositional mindfulness. Error bars indicate ±1 standard error. FFMQ: Five Facet Mindfulness Questionnaire; PSS: Perceived Stress Scale; STAI-T: State–Trait Anxiety Inventory Trait; CES-D: Center for Epidemiologic Studies Depression Scale; SD: standard deviation.

Regarding media exposure and PSS scores, a post hoc simple slope analysis showed that the perceived frequency of exposure to COVID-19-related media reports significantly predicted higher perceived stress when mindfulness was low (*β* = 0.233, *p* < 0.001) but not when mindfulness was high (*β* = 0.053, *p* = 0.177; Figure 1). Regarding the smoothness of work and psychological health outcomes, comparable simple slope analyses as detailed above revealed that views on the smoothness of work significantly predicted lower PSS, STAI-T, and CES-D scores both when mindfulness was low (PSS: *β* = −0.365, *p* < 0.001; STAI-T: *β* = −0.330, *p* < 0.001; CES-D: *β* = −0.410, *p* < 0.001) and high (PSS: *β* = −0.230, *p* < 0.001; STAI-T: *β* = −0.251, *p* < 0.001; CES-D: *β* = −0.175, *p* < 0.001; Figure 1). These data show that, even though poorer views on the smoothness of work were significantly associated with higher perceived stress, trait anxiety, and depression, regardless of mindfulness, given the significant interactions, these slopes were gentler when dispositional mindfulness was high than when it was low.

## 4. Discussion

The purposes of the present study were to uncover altered daily life behavior and its relations to psychological health associated with the COVID-19 pandemic in 2020 by involving a relatively understudied population of Japanese working adults and to examine whether and how these relationships may be moderated by dispositional mindfulness. Overall, the results not only revealed post-pandemic changes in daily life behavior but also suggested the protective roles of mindfulness for psychological health.

### 4.1. COVID-19-Related Changes in Daily Life Behavior

The present data revealed a clear trend showing that the COVID-19 pandemic altered the daily life behavior of the studied sample, in terms of both time use and their self-evaluation of life behavior. Regarding the participants’ time use, the observed post-pandemic changes involved an increase in the time spent at home and engaging in remote work, an increase in the time spent using a PC/smartphone/social media, and a decrease in the time spent outdoors and working at an office. These changes are parallel to the period of the initial state of emergency declared by the Japanese government in April 2020, which requested people to stay at home except when necessary for living everyday life ([19]; see also [14]). In fact, the mean screen time in Japanese adults 40–69 years of age was 2.3 and 2.2 h per day in 2011 and 2013, respectively [52]. The self-evaluation of daily life behavior also showed apparent changes, including increased anticipation of COVID-19 infection, increased perceived frequency of exposure to pandemic-related media reports, and a decreased degree to which the participants perceived their work to be going well. These trends are consistent with the findings from an analysis of Japanese texts posted on social media around the beginning of the pandemic [27] and suggest that such self-evaluations were held for about half a year after the COVID-19 pandemic began to influence the daily lives of the participants. Taken together, these findings suggest that the pandemic significantly altered the participants’ life behavior as measured by how they used time, as well as how they subjectively evaluated life routines, including measures directly related to COVID-19. The post-pandemic measures on time use and evaluation of life behavior were also associated with variables including household income level and the number of people per household, suggesting that the characteristics of participants such as family status are also relevant to the daily life behavior during the pandemic.

### 4.2. Altered Daily Life Behavior and Psychological Health

Consistent with our hypothesis, a large portion of variables on COVID-19-related daily life behavior were significantly associated with psychological health outcomes, especially when post-pandemic measures were considered. Regarding time use, an increase in the time spent at home and using a smartphone/social media was associated with worse psychological health, such as higher perceived stress. More apparently, self-evaluations of life behavior including the anticipation of infection, media exposure, the smoothness of work, the smoothness of nonwork activities, etc., were significantly related to psychological health. These correlations are consistent with preceding psychological studies related to the COVID-19 pandemic reporting that increases in screen time [14], massive pandemic-related information from mass media [25,26], and remote work [15], as well as worse employment status [16], have detrimental effects on psychological health. Overall, our data obtained from a Japanese sample are consistent with those from preceding studies and suggest that the self-evaluation of daily life behavior and time use were significantly associated with psychological distress during the pandemic period. However, we also found that differences in the self-evaluation of life behavior between the pre- and post-pandemic periods showed significant correlations with psychological health. Therefore, not only post-pandemic daily life behavior but also the degrees of pandemic-related changes in subjective evaluation of life behavior can impact psychological health.

### 4.3. Protective Role of Mindfulness for Psychological Health

The moderating effects of dispositional mindfulness were observed in multiple regression analyses, regarding measures on the self-evaluation of COVID-19-related life behavior. Specifically, perceived frequent exposure to COVID-19-related media reports significantly predicted higher perceived stress when mindfulness was relatively low but not when mindfulness was relatively high. Consistently, poorer views toward how well work was going were less predictive of higher perceived stress, trait anxiety, and depression when mindfulness was relatively high than when it was low, even though these views were significantly associated with lower psychological health, regardless of mindfulness. Exposure to COVID-19-related information has been suggested to increase psychological distress via rumination, even though no such mediating effect was observed when mindfulness was high [53]. In addition, higher dispositional mindfulness has been suggested to be associated with reduced emotional reactivity when facing a negative stressor [54]. Considering these preceding studies, the present data are consistent with our hypothesis and suggest that individuals high in mindfulness are better at refraining from overreacting or coping with emotional reactions in adaptive ways to reduce increases in perceived stress, even if they are frequently exposed to pandemic-related media reports. In addition, those high in mindfulness appear to have coped with negative emotions in better ways to help prevent the deterioration of their psychological health, even when facing reduced smoothness in their work. These results are also consistent with the data obtained to date during the COVID-19 pandemic that point to the desirable effects of mindfulness-based interventions [31,32], as well as associations between mindfulness tendencies and psychological health in different nonclinical populations during the pandemic period [33,34,55]. The present data add novel empirical support to the literature in an understudied population of Japanese working adults and suggest that dispositional mindfulness can serve as a good protective factor for psychological health, regardless of habitual mindfulness or meditation practice. These results obtained from a Japanese population also seem to have significance because the abovementioned psychological disposition comparable to mindfulness originated in Japanese tradition [35,36], even though whether and to what extent other cultures may have similar psychological dispositions are yet to be explored.

On the other hand, no moderating effects of mindfulness supporting our hypothesis were observed for measures regarding time use, even though the time spent engaged with social media significantly predicted higher anxiety and depression. These data indicate that the protective roles of mindfulness may be more apparent for subjective evaluation of life status than for relatively objective measures such as how individuals used time. Longitudinal studies lasting for years may further verify these possibilities.

### 4.4. Limitations

Despite the novel findings and perspectives obtained in the present study, a major limitation of the study concerns the retrospective nature of the survey, because all data were collected approximately half a year after the COVID-19 pandemic had started to influence the participants’ daily lives. Although it was practically difficult to collect data from the same participants before the pandemic period, the measures obtained for the pre-pandemic period in the present study are retrospective and may well involve a recall bias [19,50]. Additionally, stress and anxiety during the pandemic period have been suggested to impact cognitive processes such as memory functioning [56]. Therefore, during these uncertain periods, retrospective reports of the participants may not precisely reflect their psychological status several months before. To evaluate the psychological impacts of the COVID-19 pandemic and changes in daily life behavior in a timely manner, it would have been more desirable if the data were collected immediately before and after the start of the pandemic. Another major limitation relates to the use of original COVID-19-related questions. These questions were categorized into time use and self-evaluation of daily life behavior, parallel to the governmental survey conducted in a similar period; however, these items were created based on a preliminary survey with a small sample size, with no validation procedures conducted before the main survey. These bottom-up approaches seem effective during the relatively early periods of the pandemic when, for example, Japanese versions of COVID-19-related psychological scales were not yet developed [38]. Nevertheless, a more top-down approach such as using conventional scales on life routines and/or activities may have been a promising alternative in terms of, for example, directly comparing the present data with those obtained from other populations before the pandemic. Additionally, in the present sample, the number of participants assigned to each age group was not controlled for, such that approximately 80% of the participants were in their forties and fifties. Even though age was controlled in the multiple regression models, it would be preferable to assign the same numbers of participants to each age group, to allow for comparisons between age groups. In addition, whereas the present sample involved Japanese workers with a wide range of employment statuses, whether the same trends would be observed for different nonworker populations remains unknown. The focus should be placed on more specific clinical or nonclinical populations, considering the fact that mindfulness and meditation have been suggested to be beneficial for people with various psychosomatic problems during the COVID-19 pandemic [57].

## 5. Conclusions

To conclude, the present study uncovered not only changes in the daily life behavior associated with the COVID-19 pandemic in 2020 by involving Japanese working adults but also demonstrated the protective roles of dispositional mindfulness for psychological health status. These data contribute to the literature on the impact of the COVID-19 pandemic on mental health [14,15,16,19,20,21,22,23,24,25,26,27,28] and on the effects of mindfulness on maintaining psychological health during the pandemic period [31,32,33,34]. We obtained these data from an understudied population of working adults in Japan, which is a country that has historical traditions of mind–body practices relevant to mindfulness [35,36,37]. The data also suggest that dispositional mindfulness serves as a good protective factor for psychological health in this population, regardless of habitual mindfulness or meditation practice. Further studies that involve better comparisons between different occupations, levels of education, etc., are expected to better uncover how mindfulness can play a protective role in psychological health during challenging times and help propose more optimal interventions.

## Figures and Tables

**Table 1:** Basic characteristics of the participants (N = 944).

Characteristics of Participants	Proportion (%)
Sex	
Male	68.96%
Female	31.04%
Region	
Hokkaidō	3.92%
Tōhoku	4.34%
Kantō	41.95%
Chūbu	17.16%
Kansai (Kinki)	18.54%
Chūgoku	5.72%
Shikoku	2.33%
Kyūshū & Okinawa	6.04%
Marital status	
Unmarried	33.26%
Married	57.52%
Bereaved	0.21%
Divorced	9.00%
Employment status	
Self-employed/family worker	13.67%
Full-time worker	68.96%
Part-time worker	16.00%
Others	1.38%
Household income level	
Below JPA 2,000,000 Japanese	7.84%
Between JPA 2,000,000 and JPA 4,990,000	29.56%
Between JPA 5,000,000 and JPA 8,990,000	38.24%
Above JPA 9,000,000	24.36%
N people per household	
1 (Min)	22.78%
2	21.61%
3	24.58%
4	22.78%
5	6.04%
6	1.59%
7 (Max)	0.64%
Daily practice of mindfulness/meditation	
Yes	8.47%
No	91.53%

**Table 2:** Results of coronavirus disease 2019 (COVID-19)-related daily life behavior and pre- and post-pandemic comparisons.

Daily Life Behavior		Pre-Pandemic	Post-Pandemic	Pre–Post Comparison	
		M (*SD*)	M (*SD*)	*t* (*p*)	Cohen’s *d*
	Spent at home	7.58 (3.84)	8.70 (4.54)	11.123 (<0.001 ***)	0.27
	Spent indoors other than at home	6.94 (3.91)	6.17 (4.05)	−8.971 (<0.001 ***)	−0.19
	Spent outdoors	3.30 (3.33)	2.89 (3.16)	−8.229 (<0.001 ***)	−0.13
	Sleeping	6.54 (1.12)	6.58 (1.15)	2.397 (0.017 *)	0.04
Time use	Using a PC	4.02 (3.22)	4.18 (3.28)	3.452 (<0.001 ***)	0.05
(hours per day)	Using a smartphone	1.72 (1.49)	1.88 (1.63)	6.354 (<0.001 ***)	0.11
	Engaging with social media	0.59 (0.73)	0.63 (0.81)	3.196 (0.0014 **)	0.05
	Engaging in work at home	0.52 (1.77)	1.50 (2.89)	11.268 (<0.001 ***)	0.41
	Engaging in work at places other than at home	5.47 (3.99)	4.89 (4.00)	−7.319 (<0.001 ***)	−0.15
	Engaging in activities other than work	1.44 (1.44)	1.34 (1.38)	−3.621 (<0.001 ***)	−0.07
	Anticipation of infection	3.35 (2.56)	5.97 (2.48)	24.974 (<0.001 ***)	1.04
	Media exposure	3.99 (2.63)	6.22 (2.26)	21.741 (<0.001 ***)	0.91
	Well-regulated life	6.07 (2.28)	6.26 (2.24)	3.615 (<0.001 ***)	0.08
Self-evaluation	Enough sleep	6.02 (2.17)	6.13 (2.22)	2.855 (0.004 **)	0.05
	Smoothness of work	6.00 (2.13)	5.55 (2.25)	−7.881 (<0.001 ***)	−0.21
	Smoothness of nonwork activities	5.52 (2.30)	5.15 (2.32)	−5.535 (<0.001 ***)	−0.16
	Communication at work	6.03 (2.06)	5.67 (2.14)	−7.020 (<0.001 ***)	−0.17
	Communication with family	6.29 (2.16)	6.38 (2.23)	2.057 (0.040 *)	0.04

*: *p* < 0.05; **: *p* < 0.01; ***: *p* < 0.001.

**Table 3:** Scores from psychological scales and correlation coefficients between scales.

Psychological Scale	M (*SD*)	Correlation Coefficient (*r*)							
		1	2	3	4	5	6	7	8
1. FFMQ total	120.24 (13.07)	―							
2. Observing	20.60 (5.65)	0.259 ***	―						
3. Describing	24.07 (5.13)	0.792 ***	0.237 ***	―					
4. Acting with awareness	28.62 (5.50)	0.549 ***	−0.451 ***	0.263 ***	―				
5. Nonjudging	27.43 (5.53)	0.353 ***	−0.603 ***	0.065 *	0.657 ***	―			
6. Nonreactivity	19.51 (4.68)	0.549 ***	0.499 ***	0.444 ***	−0.161 ***	−0.310 ***	―		
7. PSS total	27.95 (7.56)	−0.610 ***	0.011	−0.484 ***	−0.395 ***	−0.346 ***	−0.314 ***	―	
8. STAI-T total	46.41 (10.70)	−0.589 ***	0.096 **	−0.447 ***	−0.463 ***	−0.407 ***	−0.246 ***	0.780 ***	―
9. CES-D total	16.03 (10.75)	−0.508 ***	0.129 ***	−0.359 ***	−0.469 ***	−0.377 ***	−0.186 ***	0.623 ***	0.787 ***

*: *p* < 0.05; **: *p* < 0.01; ***: *p* < 0.001.

**Table 4:** Correlation coefficients between characteristics of participants and COVID-19-related daily life behavior.

Daily Life Behavior		Age	Household Income Level	N People per Household
	Spent at home	−0.019	−0.102 **	−0.094 **
	Spent indoors other than at home	0.041	0.145 ***	0.084 *
	Spent outdoors	0.003	0.036	<0.001
	Sleeping	−0.115 ***	−0.087 **	−0.016
Time use	Using a PC	0.025	0.099 **	−0.099 **
(hours per day)	Using a smartphone	−0.265 ***	−0.036	0.063
	Engaging with social media	−0.201 ***	0.022	0.054
	Engaging in work at home	−0.065 *	0.129 ***	−0.062
	Engaging in work at places other than at home	0.020	0.047	0.037
	Engaging in activities other than work	−0.092 **	0.070 *	−0.077 *
	Anticipation of infection	−0.046	0.046	0.075 *
	Media exposure	0.039	0.099 **	0.059
	Well-regulated life	0.061	0.105 **	0.078 *
Self-evaluation	Enough sleep	0.002	0.063	0.040
	Smoothness of work	−0.012	0.213 ***	0.138 ***
	Smoothness of nonwork activities	−0.087 **	0.173 ***	0.030
	Communication at work	0.006	0.113 ***	0.091 **
	Communication with family	−0.002	0.204 ***	0.239 ***

*: *p* < 0.05; **: *p* < 0.01; ***: *p* < 0.001.

**Table 5:** Correlation coefficients (*r*) between COVID-19-related daily life behavior and total scores from psychological scales.

Daily Life Behavior		Pre–Post Difference-Based Correlation				Post-Pandemic-Based Correlation			
		FFMQ	PSS	STAI-T	CES-D	FFMQ	PSS	STAI-T	CES-D
	Spent at home	0.048	0.026	0.005	−0.015	0.009	0.068 *	0.039	0.028
	Spent indoors other than at home	−0.041	−0.023	−0.004	−0.006	−0.018	−0.076 *	−0.038	−0.023
	Spent outdoors	−0.025	−0.019	−009	−0.047	−0.014	−0.047	−0.027	0.008
	Sleeping	0.003	0.009	0.008	−0.003	−0.014	−0.031	−0.022	−0.037
Time use	Using a PC	0.035	0.011	−0.030	−0.041	0.007	0.008	0.031	0.021
(hours per day)	Using a smartphone	0.002	0.053	0.012	0.005	−0.152 ***	0.115 ***	0.136 ***	0.167 ***
	Engaging with social media	−0.022	0.043	0.032	0.044	−0.043	0.087 **	0.134 ***	0.185 ***
	Engaging in work at home	0.013	−0.001	−0.013	−0.016	0.011	0.027	0.030	0.037
	Engaging in work at places other than at home	−0.034	−0.022	−0.028	0.005	−0.036	0.002	−0.014	−0.035
	Engaging in activities other than work	−0.010	−0.042	−0.046	−0.047	0.069 *	−0.085 **	−0.046	−0.035
	Anticipation of infection	0.019	0.056	0.042	0.002	−0.029	0.131 ***	0.133 ***	0.115 ***
	Media exposure	0.029	0.071 *	0.038	−0.001	0.057	0.092 **	0.071 *	0.060
	Well-regulated life	0.036	−0.090 **	−0.039	−0.024	0.224 ***	−0.244 ***	−0.252 ***	−0.234 ***
Self-evaluation	Enough sleep	0.057	−0.046	−0.070 *	−0.094 **	0.158 ***	−0.218 ***	−0.252 ***	−0.224 ***
	Smoothness of work	−0.066 *	−0.098 **	−0.061	−0.090 **	0.187 ***	−0.387 ***	−0.389 ***	−0.376 ***
	Smoothness of nonwork activities	−0.059	−0.049	−0.015	−0.001	0.161 ***	−0.264 ***	−0.255 ***	−0.192 ***
	Communication at work	−0.019	−0.087 **	−0.015	−0.044	0.277 ***	−0.341 ***	−0.334 ***	−0.341 ***
	Communication with family	0.048	−0.050	−0.043	−0.063	0.272 ***	−0.270 ***	−0.344 ***	−0.341 ***

*: *p* < 0.05; **: *p* < 0.01; ***: *p* < 0.001.

**Table 6:** Results of hierarchical multiple regression analyses to examine the moderating roles of mindfulness (*β* coefficients).

Independent Variables		PSS			STAI-T			CES-D	
	Step 1	Step 2	Step 3	Step 1	Step 2	Step 3	Step 1	Step 2	Step 3
Step 1									
Sex	0.077 *	0.086 **	0.098 ***	0.062	0.073 **	0.079 **	−0.040	−0.031	−0.013
Age	−0.052	−0.007	−0.004	−0.050	0.007	0.010	−0.111 **	−0.049	−0.039
Unmarried	0.073	0.048	0.042	0.190 **	0.172 ***	0.169 ***	0.148 *	0.137 **	0.128 **
Married	0.057	0.026	0.027	0.115	0.091 *	0.093 *	0.059	0.046	0.050
Bereaved	0.020	0.018	0.020	0.018	0.015	0.015	−0.022	−0.024	−0.028
Full-time worker	−0.032	0.007	0.010	−0.008	0.022	0.024	0.006	0.041	0.040
Part-time worker	−0.001	0.009	0.001	0.016	0.027	0.022	0.059	0.073 *	0.064
Household income level	−0.161 ***	−0.052	−0.055 **	−0.181 ***	−0.074 **	−0.078 **	−0.146 ***	−0.047	−0.059 *
N people per household	−0.010	−0.007	−0.013	−0.014	−0.015	−0.019	−0.029	−0.031	−0.033
Daily practice of mindfulness/meditation	−0.008	−0.022	−0.020	−0.029	−0.035	−0.036	−0.068 *	−0.068 **	−0.067 **
Step 2									
Time spent at home		0.007	<0.001		−0.029	−0.032		−0.026	−0.032
Time spent using a smartphone		−0.025	−0.026		−0.006	−0.007		0.022	0.027
Time spent engaged with social media		0.020	0.022		0.062 *	0.064 *		0.103 ***	0.111 ***
Anticipation of infection		0.028	0.030		0.061 *	0.066 *		0.054	0.065 *
Media exposure		0.142 ***	0.143 ***		0.101 **	0.098 **		0.088 **	0.077 *
Smoothness of work		−0.293 ***	−0.298 ***		−0.286 ***	−0.290 ***		−0.288 ***	−0.293 ***
FFMQ total		−0.554 ***	−0.530 ***		−0.520 ***	−0.511 ***		−0.431 ***	−0.422 ***
Step 3									
FFMQ total × Time spent engaged with social media			−0.011			−0.017			0.035
FFMQ total × Anticipation of infection			0.048			−0.004			0.019
FFMQ total × Media exposure			−0.097 ***			−0.021			−0.027
FFMQ total × Smoothness of work			0.085 ***			0.050 *			0.148 ***
*R* ^2^	0.052 ***	0.492 ***	0.504 ***	0.076 ***	0.484 ***	0.487 ***	0.078 ***	0.408 ***	0.428 ***
Δ*R*^2^		0.440 ***	0.012 ***		0.408 ***	0.004		0.330 ***	0.020 ***

*: *p* < 0.05; **: *p* < 0.01; ***: *p* < 0.001.

## Data Availability

The data presented in this study are available on request from the corresponding author.
